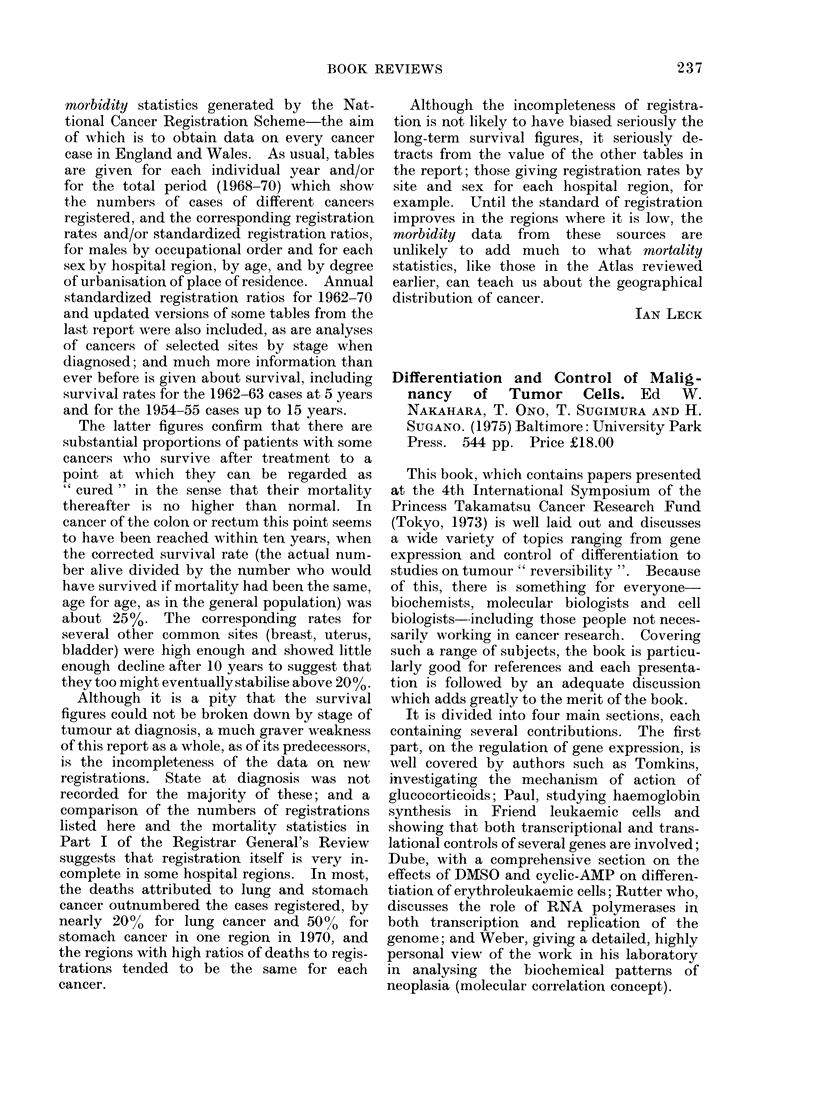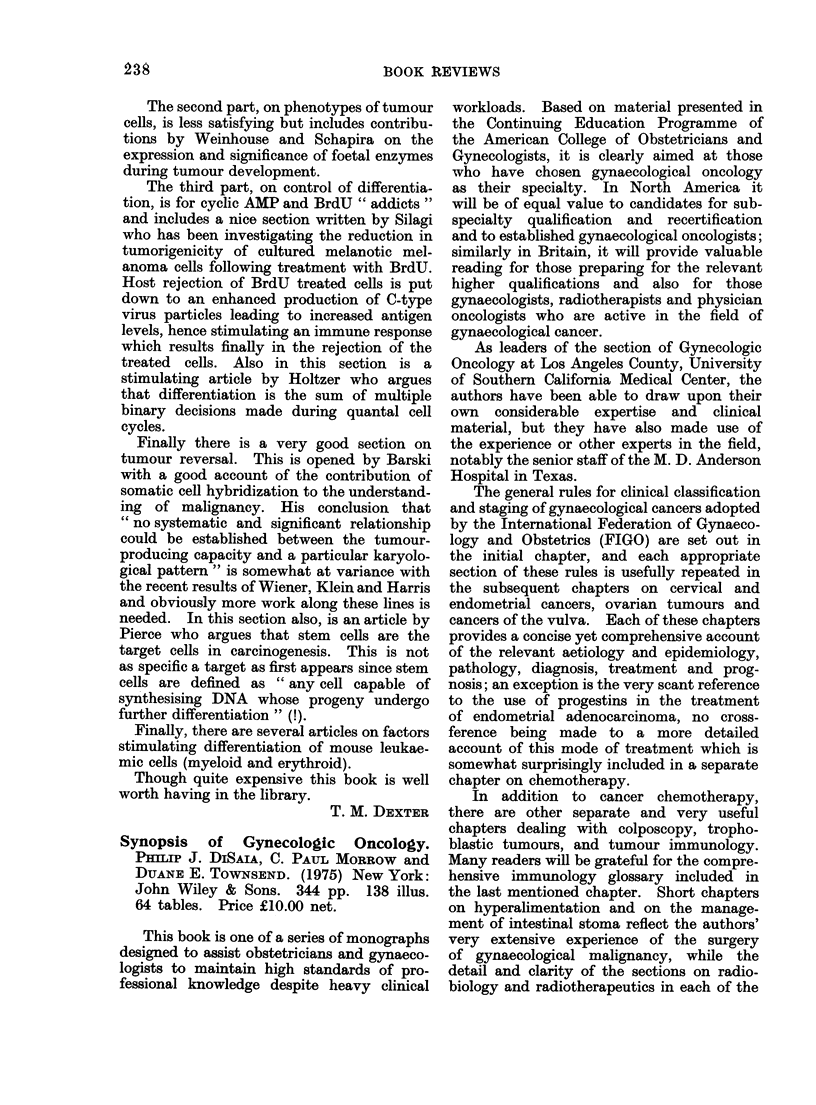# Differentiation and Control of Malignancy of Tumor Cells

**Published:** 1976-02

**Authors:** T. M. Dexter


**Differentiation and Control of Malignancy of Tumor Cells.** Ed W. NAKAHARA, T. ONO, T. SUGIMURA AND H. SUGANO. (1975) Baltimore: University Park Press. 544 pp. Price £18.00

This book, which contains papers presented at the 4th International Symposium of the Princess Takamatsu Cancer Research Fund (Tokyo, 1973) is well laid out and discusses a wide variety of topics ranging from gene expression and control of differentiation to studies on tumour " reversibility ". Because of this, there is something for everyone—biochemists, molecular biologists and cell biologists—including those people not necessarily working in cancer research. Covering such a range of subjects, the book is particularly good for references and each presentation is followed by an adequate discussion which adds greatly to the merit of the book.

It is divided into four main sections, each containing several contributions. The first part, on the regulation of gene expression, is well covered by authors such as Tomkins, investigating the mechanism of action of glucocorticoids; Paul, studying haemoglobin synthesis in Friend leukaemic cells and showing that both transcriptional and translational controls of several genes are involved; Dube, with a comprehensive section on the effects of DMSO and cyclic-AMP on differentiation of erythroleukaemic cells; Rutter who, discusses the role of RNA polymerases in both transcription and replication of the genome; and Weber, giving a detailed, highly personal view of the work in his laboratory in analysing the biochemical patterns of neoplasia (molecular correlation concept).

The second part, on phenotypes of tumour cells, is less satisfying but includes contributions by Weinhouse and Schapira on the expression and significance of foetal enzymes during tumour development.

The third part, on control of differentiation, is for cyclic AMP and BrdU " addicts " and includes a nice section written by Silagi who has been investigating the reduction in tumorigenicity of cultured melanotic melanoma cells following treatment with BrdU. Host rejection of BrdU treated cells is put down to an enhanced production of C-type virus particles leading to increased antigen levels, hence stimulating an immune response which results finally in the rejection of the treated cells. Also in this section is a stimulating article by Holtzer who argues that differentiation is the sum of multiple binary decisions made during quantal cell cycles.

Finally there is a very good section on tumour reversal. This is opened by Barski with a good account of the contribution of somatic cell hybridization to the understanding of malignancy. His conclusion that " no systematic and significant relationship could be established between the tumour-producing capacity and a particular karyological pattern " is somewhat at variance with the recent results of Wiener, Klein and Harris and obviously more work along these lines is needed. In this section also, is an article by Pierce who argues that stem cells are the target cells in carcinogenesis. This is not as specific a target as first appears since stem cells are defined as " any cell capable of synthesising DNA whose progeny undergo further differentiation " (!).

Finally, there are several articles on factors stimulating differentiation of mouse leukaemic cells (myeloid and erythroid).

Though quite expensive this book is well worth having in the library.

T. M. Dexter